# Numerical Cognition after Brain Injury: Is There a Relationship between Subitizing and Arithmetical Abilities?

**DOI:** 10.3390/brainsci13030381

**Published:** 2023-02-22

**Authors:** Esther Gosling, Nele Demeyere, Ann Dowker

**Affiliations:** 1 St. Hilda’s College, Oxford University, Oxford OX4 1DY, UK; 2 Department of Experimental Psychology, Oxford University, Oxford OX2 6GG, UK

**Keywords:** numerical cognition, arithmetic, dyscalculia, post-stroke patients, subitizing

## Abstract

Subitizing is the ability to enumerate small quantities efficiently and automatically. Counting is a strategy adopted for larger numerosities resulting in a near linear increase in response time with each increase in quantity. Some developmental studies suggest that being able to subitize efficiently may be a predictor of later arithmetical ability. Being able to enumerate small quantities efficiently may be necessary for at least some aspects of arithmetical skill and understanding to develop. According to this view, arithmetic ability ultimately depends upon subitizing. If this were the case, when acquired brain injury results in impaired performance on subitizing tasks, mathematical performance may also be impaired. The following study tested eleven healthy control participants and nine chronic patients with acquired brain injury on tasks focused on visual enumeration, addition and multiplication to explore a potential relationship between subitizing ability and calculation performance. No overall correlations were found between subitizing and addition or multiplication speed. However, a very clear subitizing impairment was found in two patients who then demonstrated very different levels of preserved addition skills. The dissociations found and the large inter-individual variability supports a more componential view of arithmetical ability.

## 1. Introduction

A human’s ability to enumerate visual stimuli involves not only verbal counting, but also counts on two nonverbal quantity recognition processes: the Approximate Number System (ANS) [1]), which involves the approximate estimation of quantities above three or four items, and subitizing. Subitizing (a word developed from the Latin meaning for suddenly) is a term referring to an ability to make rapid yet accurate number judgments about small sets of stimuli presented in the visual field. There have been some studies of small-numerosity judgements and how they may differ from larger-numerosity judgements since at least the 19th century [2] and several relatively early studies, e.g., [3], show that the speed of small-numerosity judgements is much less affected by increases in number size than is the case for larger-numerosity judgements. The actual term ‘subitizing’, however was first coined by Kaufman et al. in 1949 [4].

Though there are debates about the exact definition of subitizing, it is usually considered as meaning an exact but language-independent representation of small quantities, which is present in babies before they can speak and is often thought to be innate and to form all or part of the basis for subsequent numerical ability.

There are some disagreements about the extent of the subitizing range. Kaufman et al. [4] proposed that it extended to six items. Mandler and Szebo [5] suggested that it extended just to three items. Trick and Pylyshyn [6] suggested that it extended to four items. Starkey and Cooper [7] proposed a subitizing range of three in infants and young children and five in adults. The addition of an item within this range only increases the response time by a small amount. This small increase has been reported to range between 50 ms and 80 ms e.g. [5]. However, when the number of items surpasses the limit of subitizing, adults will typically adopt a counting strategy. In contrast to subitizing, this process results in a near linear increase in the response time with each item added to the visual scene. After four items, the response time has been reported to increase by 250–350 ms per item [6,8,9,10].

It is believed by some that different underlying processes support subitizing and counting (though see, e.g., [11] for an opposite view). According to such theories, subitizing is a result of rapid parallel processing whereas counting is a much slower serial process. There are several different models that present a dual process for enumeration. Some neuropsychological evidence supports a dissociation between subitizing and counting. Damage to the left posterior occipital cortex, bilateral lateral occipital and right superior frontal cortices were found to be associated with enumeration in the subitizing range whereas damage to the left intraparietal sulcus was found to be associated with counting deficits. This suggests that subitizing and counting rely, at least to some extent, on different processes and neural areas [12,13]. 

Despite the findings mentioned above, it is likely that the two processes are still linked in some way. There is substantial evidence that counting relies on the accurate development of subitizing. This may be due to some underlying neuropsychological similarities or the foundational nature of abilities that support numerical representations, one of which is an innate capacity to represent small numerosities. This capacity is demonstrated in subitizing tasks; and Butterworth [14] proposed that such subitizing ability lies at the foundation of mathematical ability. Young children, through habituation and transformation tasks, have been shown to recognize and discriminate small numbers of objects [15], highlighting the potential innateness of subitizing. There are debates about whether subitizing or the ANS appears earliest in infancy and is most crucial to numerical development [1]); however, it is now generally considered that both are present at or near the beginning and are applied to different sizes of quantities: subitizing to smaller quantities and the ANS to larger quantities. The ability to subitize appears to develop before the ability to count. In support, it has been shown that children are not able to count amounts they cannot subitize [16]. 

Subitizing has been linked to the development of various aspects of mathematical ability [14,17,18,19,20]. 

For example, Desoete and Gregoire [21] found subitizing ability to be predictive of later mathematical performance. They administered standardized mathematical assessments to 82 children at the end of kindergarten. They then re-assessed the mathematical achievement of the children during 1st grade. Below average subitizing ability was predictive of low achievement in mathematics. Hannula-Sormunen et al. [22] found that preschool children’s spontaneous focusing on numerosity, subitizing and counting skills all predicted their mathematical performance seven years later. Sowinski and LeFevre [23] found that subitizing correlated with arithmetic fluency in typical adults.

In line with these findings, problems in subitizing have been identified in some children and adults with dyscalculia. Developmental dyscalculia involves severe specific arithmetical disabilities that are present from the beginning and is considered by some researchers to result from a core deficit in number sense [24,25,26,27]. A deficit in subitizing may reflect or cause a deficit in number sense thus being central to impairments in arithmetic. For example, Reeve and Reynolds [28] found 6% of a randomly selected group of infants did not demonstrate the ability to subitize. The infants were tracked and tested longitudinally. When tested as schoolchildren, it was found that those who did not show evidence of subitizing in infancy were much slower at reading three-digit numbers compared to the rest of the sample. Landerl et al. [29] also reported that those with subitizing deficits were also impaired in arithmetic tasks and were slow to differentiate numbers. Similar results were obtained by Schleifer and Landerl [30].

Reigosa-Crespo et al. [31] studied 11,652 children in Havana, Cuba from 2nd to 9th grades. A total of 3.4% of this population had deficits in basic numerical capacities, including subitizing small quantities and almost all of these showed calculation difficulties. An additional 9.35% showed poor calculation, without the basic numerical deficits. Those with basic numerical difficulties differed in some important ways from the rest of the children with poor calculation differences—for example, a majority were boys and their difficulties tended to be more severe. Estevez-Perez et al. [32] followed up this study and also looked at typically developing children. They found that subitizing, verbal counting and numerical magnitude comparison all predicted an early and later acquisition of arithmetic and were impaired in children with low arithmetic achievement and with developmental dyscalculia. They also found that children with low arithmetic achievement, who were also poor at subitizing, were slower and relied more on compensatory strategies in arithmetic than apparently similarly low attainers, who showed typical subitizing ability. It is, however, possible that at least in some cases, both the subitizing weaknesses and the arithmetical difficulties may arise from some other factor, such as problems with attention or executive function. Ashkenazi et al. [33] suggested that weaknesses in pattern recognition might be involved.

If subitizing is important in the development of some or all aspects of arithmetic, one might expect that training in subitizing might improve mathematical performance [34,35]. Ozdem and Olkun [36] did find that 640 min of training over an eight-week period in conceptual subitizing skills improved second- and third-grade children’s overall mathematical performance both immediately after the end of the intervention and a semester later.

However, some studies suggest that subitizing and arithmetic are unrelated. Anobile et al. [37] found that simultaneous and sequential subitizing did not correlate with one another and that neither predicted either mental calculation or digit magnitude knowledge in primary school children. By contrast, the estimation of larger numerosities did predict children’s arithmetic [38]. (Anobile et al. [39] have further proposed that there may not be, as is often thought, just two visual numerosity processing systems: one for small numerosities (subitizing) and one for larger numerosities (estimation). They propose that there may be three such systems: one for numerosities under 4; one for high numerosities, and one for intermediate numerosities such as those between 10 and 20. They suggest that the intermediate numerosity system may depend less on attention than either the low or high numerosity system. This proposal is based both on findings that performing a distracting concurrent task impaired typical adults’ performance on numerosity judgement tasks far more for low or high numerosities than for intermediate numerosities, and on a study of a simultagnosic patient, whose ability to compare either very low or very high numerosities was seriously impaired, while his comparison of intermediate numerosities was relatively preserved.

Decarli et al. [40] found little evidence for subitizing deficits in developmental dyscalculia, and far more evidence for difficulties with the approximate estimation and comparison of larger quantities. Even those who do find a link between subitizing deficits and developmental dyscalculia have tended to find that only some individuals who are clinically diagnosed with dyscalculia present problems in subitizing. Desoete et al. [41] found that only 33% of a sample of 30 dyscalculic children, aged eight and a half, showed severe subitizing deficits. Iuculano et al. [42] studied two boys with a diagnosis of developmental dyscalculia, and found that one had problems with subitizing, and the other mainly with the ANS.

The existence and extent of links between subitizing and arithmetic are just one aspect of the larger area of the question of associations and discrepancies between different components of numerical ability. There is considerable evidence from educational, factor analytic and neuroimaging studies of typically developing children and adults for numerical cognition being made up of many different components, and for marked individual differences in the relative levels of functioning of the different components [43,44,45]. Some particularly striking evidence comes from studies of patients who have become dyscalculic as the result of brain damage. For example, patients can show selective impairments in factual, conceptual and procedural knowledge of arithmetic. The largest-scale study was probably that conducted by Cappelletti et al. [46], who investigated a variety of numerical and calculation skills in patients and controls. Patients with neurodegenerative disorders or focal brain lesions were compared to healthy patients who had not suffered any brain injury. The patients were additionally tested on a set of non-numerical semantic tests to allow for comparison across knowledge types. Results showed that all participants, even those with parietal damage, performed accurately on simple number processing tasks. However, the majority of focal patients, wherever their lesions were located, were impaired on calculation tasks (though most with neurodegenerative disorders were not). This would suggest that calculation skill is not solely reliant on number processing, supporting a more componential view of numerical ability.

Somewhat similar dissociations were found in patients suffering from the early stages of Alzheimer’s disease [47]. Within their patient sample, a variety of abilities were measured including the understanding of arithmetic facts, arithmetic procedure and the ability to understand and compare numbers. It appeared that no component of arithmetical ability was a necessary prerequisite for any other.

In the present study, a visual enumeration task and a set of addition tasks were administered to a group of neuropsychological patients, with a variety of lesion locations, as well as a control group of age-matched adults. We first tested the hypothesis that in patients and controls, there would indeed be a significant difference in reaction time for the enumeration of numerosities from one to three and of numerosities from four to eight. We also expected that reaction times for enumeration of the smaller numerosities would not correlate significantly with reaction times for the larger numerosities, in either patients or controls. If this combination of results were found, it would give support to the assumption that there is a distinction between the processes used for enumerating the smaller and the larger numerosities, which may be described as subitizing versus counting.

The main purpose of the study was to investigate whether there is a link between subitizing impairments and arithmetical difficulties in a group known to have a high risk of calculation impairments: patients with chronic acquired brain injury [46]. We predicted that, if there were such a link, it would be particularly noticeable for addition, as formal addition is usually one of the earliest arithmetical skills that children acquire and may be more directly influenced by foundational abilities such as subitizing, while later-developing arithmetical skills may build more on addition. The patients were predominantly investigated as a set of case studies, rather than a homogeneous group. It was hypothesized that if there is a strong association between subitizing and addition, then impairment in the former would be associated with impairment in the latter. It was predicted that patients who struggled with subitizing either in regard to accuracy or reaction time or both, would also show lower performance levels in addition tests. For the purposes of this study, the subitizing range is treated as extending to three items, rather than four or a larger number. This is an a priori decision, following the criteria used by Demeyere et al. [13].

A secondary aim of the study was to investigate the effects, for both patients and controls, of specific characteristics of the addition sums that might influence the load on working memory. Specifically, we predicted that sums that were classified as descending (with the second addend smaller than the first) would be easier than those classed as ascending (with the second addend larger than the first), resulting in shorter reaction times. We also predicted that the more decades that had to be bridged, the longer the reaction times would be.

## 2. Methods

### 2.1. Participants

A total of 21 adult participants took part in the study: including 9 patients and 12 controls. Three tasks were carried out: Visual enumeration, Addition, and Multiplication. All participants completed the enumeration experiment. However, not all participants completed the full Multiplication and Addition tasks due to time constraints and limited capacities of some patients.

Data were collected from all 12 of the control participants. However, one control was not included in the analysis as their addition accuracy was below the set cut-off score of 80%, leading to a final control group of 11 participants.

The age of the control group ranged from 62 to 80 years (Mean age = 65.5 years, SD = 7.2 years). The control group comprised of 4 female participants and 7 male participants. To be eligible for the control group, participants had to have no history of any neurological abnormalities. The control group was recruited through advertising from the Cognitive Neuropsychology department at the University of Oxford.

Patients: 9 patients participated in the study. The ages of the patients ranged from 32 to 80 years (M age = 57.2, SD = 15.9). The patient group consisted of 7 male patients and 2 female patients, all of whom were recruited through a voluntary panel at the Cognitive Neuropsychology Department of the University of Oxford. Eight had had strokes, and one had posterior cortical atrophy. All stroke patients were in a chronic stage (at least 9 months post stroke) and presented a wide variety of symptoms and impaired and preserved abilities.

Table 1 displays a summary of the clinical and demographic details of the patient group.

#### 2.1.1. To Give Some More Detail on Areas of Brain Damage

##### Patients with Left Hemisphere Damage

Patient P experienced damage to 100% of his left uncinate fasciculus and a majority (50–95%) of his left external capsule, planum polare, insular cortex, planum temporale, Heschl’s gyrus, frontal opercular cortex, superior longitudinal fasiculus, retolenticular part of the internal capsule and anterior corona radiata.

Patient R experienced damage to the majority (75–99%) of his left central opercular cortex, planum polare, Heschl’s gyrus, parietal opercular cortex, planum temporale, superior temporal gyrus, anterior unculate fasiculus, middle temporal gyrus (posterior division) and superior temporal gyrus (posterior division).

Patient L experienced damage to a majority of her left superior fronto-occipital fasiculus, uncinate fasiculus, external capsule, anterior and posterior limbs of the retrolenticular part of the internal capsule, fornix (cres) stria terminalis and insular cortex, and over 40% of her left cerebral peduncle and sagittal striatum.

Patient S experienced damage to over 95% of his left frontal opercular cortex and left lateral frontal gyrus pars opercularis and a majority (55–79%) of his left pars triangularis, insular cortex, external capsule, subcallosal cortex, planum polare, anterior limb of the internal capsule and paracentral gyrus.

##### Patients with Right Hemisphere Damage

Patient J experienced damage to a majority of his right frontal opercular cortex, insular cortex and central opercular cortex; over 30% of his uncunate fasiculus, external capsule and planum polare, and smaller amounts of his right inferior frontal gyrus pars operculus, anterior corona radiata, inferior frontal gyrus pars triangularis and precentral gyrus.

Patient N experienced damage to a majority (74–99%) of his right cingulum (hippocampus), intracalcarine cortex, occipital fusiform gyrus, temporal-occipital fusiform cortex, temporal fusiform cortex (posterior division), cuneal cortex, supracalcarene cortex, lingual gyrus and fornix (cres) stria terminalis

Patient M experienced damage to 55% of his right external capsule, over a third of the right posterior and anterior limbs of his right internal capsule, 22% of the reticulolenticular part of his right internal capsule, and smaller parts of his right fornix (cres) stria terminalis, superior fronto-occipital fasiculus and insular cortex.

Patient E experienced damage to 14% of his right anterior corona radiata frontal opercular cortex and less than 10% of his right superior corona radiata insular cortex, superior longitudinal fasiculus, superior fronto-occipital fasiculus, anterior limb of internal capsule and middle frontal gyrus.

##### Patient with Posterior Cortical Atrophy

Patient A did not have a localized lesion, but had posterior cortical atrophy.

### 2.2. Materials and Procedure

E-Prime software was used to deliver all tests and to record the data. For each condition, participants sat in front of a computer screen and used the keyboard to respond and record answers throughout. Due to some patients suffering from motor control problems, a keyboard response was deemed the most appropriate method.

The space bar was used to record reaction times and the numeric keypad for recording answers. Due to some patients suffering from aphasia, numerical presentation and responding was used to reduce the possibility of their aphasia affecting the expression of numerical ability. Each time the test would be loaded by the experimenter and would begin with brief instructions about the experiment that was to follow. This included an explanation of the task and how they should respond. Participants then began the test by pressing the space bar when they understood the task demands.

In each test, before the stimuli were presented, a white centered fixation cross was presented on a black screen for 1000 ms. Some participants had limitations in their ability to use the keyboard. In such cases, the participant would press the space bar to record their response time but would speak the answer aloud, allowing the experimenter to enter, via the keypad, their response.

Visual Enumeration. Each trial began with the fixation cross which was then replaced with a black screen. The area of possible presentation of the grey dots had a visual angle of 14.4°. Grey dots (visual angle of 1.4°) were randomly positioned and simultaneously presented in each trial. The number of dots varied between 1 and 9 with each block consisting of 5 trials per numerosity, thus totaling 45 trials per block. The dot display remained on the screen for an unlimited duration and would only disappear when participants made a response. Participants were instructed to enumerate the dots on the screen as quickly and accurately as possible. As soon as they felt they knew how many dots there were they pressed the space bar, which recorded their response time. Once the space bar had been pressed, a screen asking the question ‘How many?’ appeared giving participants the chance to type in their answer. This part of the response process was untimed, allowing participants to answer slowly and accurately without any form of time pressure.

The study consisted of 6 blocks, with breaks in between. The duration of the set of tasks varied depending on participants’ speed. For controls, it took about 40 min.

Addition. The addition task was made up of 2 blocks, each consisting of 64 trials. For each trial, the fixation cross was replaced with a black screen displaying an addition equation in the format of ‘[number] + [number] =’. Each number was presented in digits with the response also being recorded using digits. The numbers used in the equation ranged from 1 to 99. The experiment consisted of both ascending and descending sums and to correctly answer, they could either bridge 0, 1 or 2 decades. When the participant had worked out the answer, they pressed the space bar and entered their response using the numeric keypad, finally pressing ‘enter’ to submit their answer. Between the two blocks, a text display of a white background and black letters appeared, signaling to participants that they could take a break if required. The condition concluded with another text display indicating the condition was over.

All controls completed the maximum set of 128 trials. All patients except from patient A, L and P also completed the whole task. Patient L only completed one block of 64 trials due to fatigue and patient P only completed 49 trials due to time constraints. Patient A, due to severe visual and mathematical deficits completed a much shorter addition task. For this, a total of 14 sums were presented to her on paper and results extrapolated from this small set. Thus, it was not possible to acquire response times for patient A.

Multiplication. The Multiplication experiment consisted of one block of 64 trials. There were 8 of each timetable from 2 to 9. For each trial, after the fixation cross disappeared, a multiplication sum appeared in the format of ‘[number] × [number] =’. The equation was presented in numerical form. Participants responded, using the space bar, as quickly as they could. A cursor then appeared on a black screen prompting the participants to type in their answer. The ‘enter’ key was used to submit their answer. The condition finished with a screen announcing the end of the experiment.

Four patients completed all 64 trials, with patient A completing a shortened version of 37 trials due to time constraints. A total of 7 controls completed the multiplication task.

## 3. Results

This section will first report the data from the individual patients and how they compare with the control group, followed by some analyses of differences between the task conditions and correlations between tasks. The data from each experiment was first analyzed separately for each individual. Response times (RTs) were cleaned by removing outlier individual trial data points that fell outside three standard deviations from the mean score of each participant. Patient accuracy scores and RTs were analyzed, using Crawford’s *t*-test, to decipher the exact impairments seen in each patient relative to the control group. Related-sample Wilcoxon tests were used to explore t differences between the task condition within the patient group and the control group. Correlations between performance on different tasks were explored using Spearman’s rank correlation.

### 3.1. Patients and Controls’ Scores on Visual Enumeration and Arithmetic Measures: Descriptive Statistics

The data were first separated into small-number (presumed subitizing) and larger-number (presumed counting) data. Subitizing accuracy data was taken as the average of scores to one, two and three dots, with the subitizing slope being taken as the average step-size between response times to two dots and three dots (i.e., average RT (three dot)—average RT (two dots).

To analyze the larger-number counting, an RT slope was calculated across the counting range using a linear regression. This range was considered to involve numbers 5–8. The maximum display number of nine was excluded as responses to this display may have been affected by end guessing [6].

Table 2 gives the descriptive data for the control group’s performance in the enumeration and arithmetic tasks.

**Figure 1:**
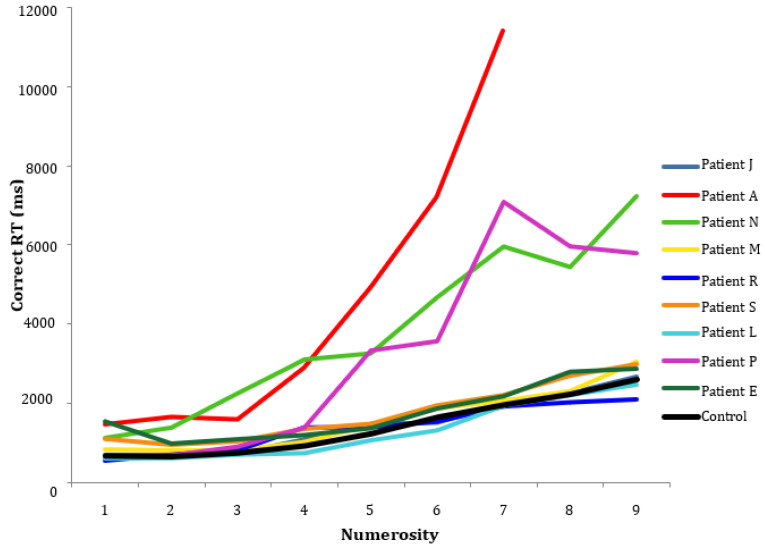
Response times of each patient and mean for control group on each numerosity.

The patients’ response time for correct responses to each numerosity, and the mean for the control group, are shown in Figure 1. As can be seen, most patients had similar response times to the control group mean, but patients P and N and especially A were slower.

**Figure 2:**
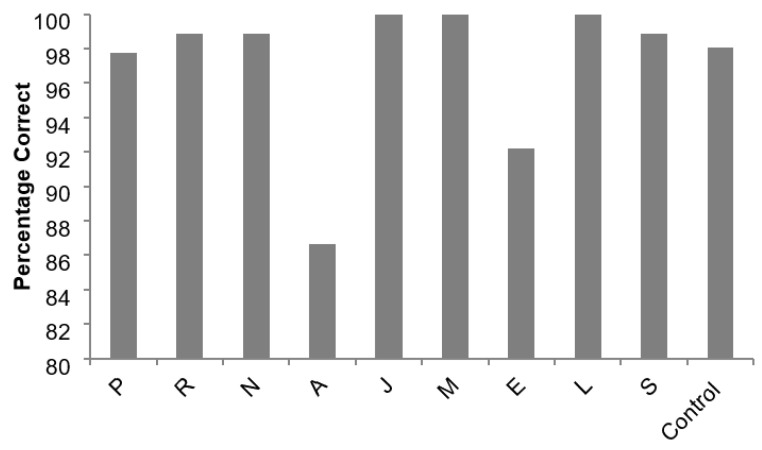
The percentage of correct responses given when enumerating 1, 2 and 3 dots given by individual patients and the mean of 11 controls.

Each patient’s percentage of correct responses for enumeration of numerosities of one to three (classed as subitizing) is shown in Figure 2, together with the mean for the controls. As can be seen, the controls and most patients performed close to the ceiling on this task, but patients A and E were less accurate.

**Figure 3:**
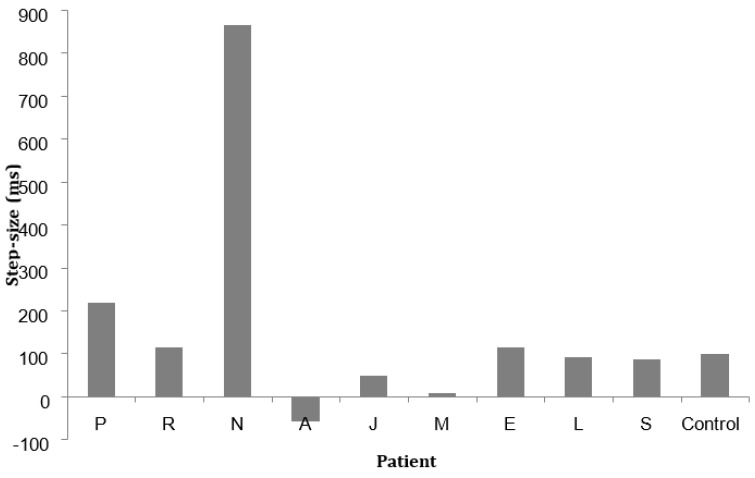
The difference in correct response times (RTs) to the enumeration of 2 and 3 dots given by individual patients and the mean of 11 controls.

Each patient’s subitizing speed step-size is given in Figure 3, together with the mean for the controls (97.21; sd. 42.58). Most patients showed similar step-size to the controls, with P and especially N showing unusually large differences for two and three dots, and A showing a difference in the reverse direction.

Table 3 shows each patient’s performance on key enumeration measures, and their accuracy scores and mean reaction times for correct answers for addition and (where given) multiplication problems.

### 3.2. Differences between Performance of Individual Patients and Controls: Statistically Significant Impairments

Using the mean and standard deviation from the control data, a normal range for each task was calculated (mean + or −2 ½ SD). To analyze the individual patient data points that fell outside this range, relative to control data, a variant of the *t*-test was used [48,49]. (A Crawford’s *t*-test was run on each of these outlying data points to decipher if the patients’ performance was significantly different to the controls. Descriptive data, from control participants and for each condition, are shown in Table 2. A summary table of the significant results and thus the impairments displayed by the patients is shown in Table 4.

### 3.3. Reaction Times to Smaller and Larger Numerosities

In the patient group, reaction times to numerosities of four or more (M = 343.24, SD = 40.90) were significantly slower than those for numerosities from one to three (M = 95.78, SD = 11.81); test statistic = −2.55, *p* = 0.011). A similar difference was found for the controls (test statistic = −3.059; *p* = 0.002). Reaction time for numerosities from one to three did not correlate significantly with reaction times to numerosities of four or more in the patient group (rho = −0.139; *n* = 9; *p* = 0.72), the control group (rho = 0.105; *n* = 11; *p* = 0.746) or the two groups combined. (rho = 0.293; *n* = 20; *p* = 0.378.)

### 3.4. Effects of the Characteristics of the Addition Sums

Further analyses within the addition condition were conducted by looking at the order of the presentation of the numbers within the sum. Each sum was labelled either Ascending (Number < Number = ?) or Descending (Number > Number = ?). In the addition test, the sums were also sorted with regards to the number of decades that had to be bridged in order to work out the answer (zero, one or two).

Sums that were classified as descending were hypothesized to be easier. This hypothesis was supported in both groups and in the two groups combined. Related-samples Wilcoxon tests were carried out in each group separately and in the two groups combined, comparing the response times in the two conditions. In the patient group, response times to ascending addition sums (M = 6055.65, SD = 8051.07) were significantly slower than response times to descending addition sums (M = 5694.6, SD = 6713.62); standardized test statistic = −3.662, *n* = 9, *p* = 0.018). In the control group, there was also a significant difference between response times to ascending addition sums (M = 2560.90, SD = 269.87) and response times to descending addition sums (M = 2411.97, SD = 230.75); standardized test statistic = −2.045, *n* = 11, *p* = 0.041. In the whole sample, there was a significant difference between response times to ascending addition sums (M = 4026.41, SD = 5523.43) and response times to descending addition sums (M = 3752.27, SD = 4344.16); standardized test statistic = −3.027, *n* = 20, *p* = 0.002.

We also investigated the possibility that the more decades that had to be bridged to gain an answer, the longer the sum would take. This hypothesis was supported in both groups and in the two groups combined. Related-sample Wilcoxon tests were carried out in each group and in the two groups combined. In the patient group, response times to addition sums that bridged a decade (M = 4431.18, SD = 4197.4) showed a borderline significant difference from those addition sums that did not bridge a decade (M = 3698.98, SD = 3765); standardized test statistic = −1.96, *n* = 9, *p* = 0.05. They were significantly faster for addition sums that bridged one decade than for addition sums that bridged two decades (M = 9612.27, SD = 15,421.07); standardized test statistic = 2.366, *n* =9, *p* = 0.018. In the control group, response times to addition sums that bridged a decade (M= 2479.24, SD = 920.55) were significantly slower than addition sums that did not bridge a decade (M = 1895.79, SD = 602.44); standardized test statistic = 3.059, *n* = 11, *p* = 0.002. They were significantly faster than those to addition sums that bridged two decades (M = 3175.43, SD = 1033.53); test statistic = −3.058, *n* = 11, *p* = 002. In the two groups combined, response times to addition sums that bridged a decade (M = 3620.02, SD = 2818.52) were significantly slower than addition sums that did not bridge a decade (M = 2625.49, SD = 2495.15), standardized test statistic = −3.472; *n* = 20; *p* = 0.002. They were significantly faster than those that bridged two decades (M = 5698.34, SD = 9472.25); standardized test statistic = 3.823, *n* = 20. *p* < 0.001.

### 3.5. Correlations between Subitizing and Addition Performance

To test the hypothesis that subitizing performance has an effect on addition performance, a Spearman’s rank correlation was used to investigate any relationships between addition and subitizing data. A Spearman’s rank correlation was used as the data set was small and the patient sample was heterogeneous: thus, a normal distribution could not be assumed. There was no significant correlation between subitizing reaction time and response time to addition sums either in the patient group (rho = 0.429; *n* = 9; *p* = 0.289), the control group (rho = 0.035; *n* = 11; *p* = 0.914) or in the two groups combined (rho = −0.12; *n* = 20; *p* = 0.641). There was also no significant correlation between subitizing speed reaction time and addition accuracy either in the patient group (rho = −0.367; *n* = 9; *p* = 0.371), the control group (rho = 0.127; *n* = 11; *p* = 0.624) or in the two groups combined (rho = −0.272; *n* = 20; *p* = 0.247). To further explore the possibility of a relationship, Spearman correlation coefficients were calculated between the subitizing speed and response time to addition sums that bridged two decades, due to the increased difficulty of this type of addition sum. Again, no significant correlation was found in the patient group (rho = 0.571; *n* = 9; *p* = 0.18), the control group (rho = −0.098; *n* = 11; *p* = 0.701), in the two groups combined (rho = −0.12; *n* = 20; *p* = 0.641) or overall. Results were similar for correlation between the subitizing speed and accuracy for addition sums that bridged two decades. These did not correlate significantly in the patient group (rho = −0.611; *n* = 8; *p* = 0.108), the control group (rho = −0.068; *n* = 12; *p* = 0.845) or in the two groups combined (rho = 0.242; *n* = 20; *p* = 0.304).

### 3.6. Multiplication

The results for multiplication will be described quite briefly, as only five patients took the test. These were patients R (accuracy score 87.5%; mean correct response time 4082.84 ms); N (accuracy score 89.06%; mean correct response time 2055.43 ms); A (accuracy score 62.16%; mean correct response time 29,589.82 ms); J (accuracy score 100%; mean correct response time 931.56 ms); and M (accuracy score 98.43%; mean correct response time 1739.59 ms). Seven controls took the test, with a mean accuracy score of 89.29% (s.d. 3.87) and a mean correct response time of 1775,28 ms (s.d. 604.48).

The subitizing reaction time did not correlate significantly with the multiplication response time in the patient group (rho = −0.2; *n* = 5; *p* = 0.747); the control group (rho = 0.286; *n* = 7; *p* = 0.535 or the two groups combined (rho = 0.112; *n* = 12; *p* = 0.729). The subitizing reaction time did not correlate significantly with multiplication accuracy in the patient group (rho = 0.2; *n* = 5; *p* = 0.747); the control group (rho = −0.543; *n* = 7; *p* = 0.208) or the two groups combined (rho = 0.34; *n* = 12; *p* = 0.279).

## 4. Discussion

### 4.1. Some Support for a Componential View of Arithmetical Ability

The view that arithmetical ability is componential rather than unitary [43,44,45] is supported by both the patient data and control data from this study. Arithmetical ability does not appear to be a single characteristic, instead made up of different abilities invariably reliant on each other, despite the potential for correlations to be found between certain abilities. It is likely that each component of arithmetical ability is potentially, functionally independent and that the components do not invariably depend on each other.

Firstly, the basic assumption of the study, that there would be a distinction between small-number enumeration (subitizing) and larger-number enumeration (counting), was strongly supported. Not only did RTs differ significantly between small number enumeration and larger-number enumeration, but there was no significant correlation between the two, supporting our decision to treat subitizing as a specific numerical process.

Moreover, within the patient and control samples separately and combined, no correlations were found between the subitizing speed and performance on addition sums, or so far as could be concluded from the reduced samples, between the subitizing speed and performance on multiplication problems. Lack of overall correlations could potentially reflect the relatively small samples. However, there were also no obvious links or relationships between deficits shown within the patient group. There were no clear patterns of deficits across patients as different patients displayed a variety of different impairments. Subitizing speed deficits did not appear to be consistently associated with specific problems with addition. For example, despite showing impaired subitizing performance, patient N and patient E performed within the normal range on addition sums. Patient N appeared to adopt a counting strategy for all numerosities as there was a steady increase in response time with an increase in dots. He did not appear to be able to subitize yet did not display impaired arithmetic. This is not congruent with the idea that subitizing ability is essential for arithmetical ability.

By contrast, patient A and patient P both showed deficits in subitizing performance and problems with addition. However, both patients showed very general impairments as they performed outside the normal range on the majority of the tasks and conditions. Specifically, patient A appears to have a more general cognitive impairment. The pervasive nature of the deficits observed in these two patients makes it difficult to draw any conclusions about specific impairments and any relationships between certain abilities that there may be. As further evidence for lack of a consistent relationship between patients’ impairments, patient S displayed normal subitizing ability yet struggled with addition sums in the majority of the conditions. The lack of a consistent relationship between impairments in patients points towards a more componential view of arithmetical ability,), and in particular would suggest a functional independence between mathematical performance and subitizing ability. This is in line with a number of previous findings [1,46,47,50], though some have found stronger associations between deficits in subitizing and deficits in arithmetic [14,51,52]. 

There does not seem to be a strong relationship with the side or nature of the brain injury and the level or type of impairment found, except that patient A, who had bilateral brain dysfunction, had the most severe and widespread deficits. Patients P and R had very similar brain lesions, but P had widespread deficits in subitizing and addition, while R had no deficits in these areas and only a deficit in multiplication RT (these two patients will be discussed further in Section 4.3). The patients without any deficits in the numerical abilities studied here included patient L with left hemisphere damage and patients J and M with right hemisphere damage. It could be argued that, as patient L was left-handed, she might have had atypical hemisphere specialization; however, this appears unlikely, as her left hemisphere lesion had resulted in literacy deficits.

Though the present study investigated several different numerical abilities, it did not investigate all possible ones, and future studies should investigate a wider range of abilities, especially those that have been proposed as foundations for mathematical development, such as the ANS [1], symbolic numerical processing [53], and counting concepts, e.g., [54,55], especially cardinality, which has been found to be particularly strongly predictive of early arithmetical development in young children [56] It is quite likely that testing the latter would result in ceiling effects for most participants, but it is still desirable to investigate it.

### 4.2. The Potential Role of Individual Differences

The rejection of the proposed hypothesis of the relationship between subitizing and arithmetic in patients may be premature. The variation in deficits seen in patients, and between patients, could be explained at least in part as due to pre-existing individual differences in mathematical ability. Despite suffering from a similar lesion to patient R, patient P displayed a much larger set of impairments. He was generally significantly slower in all conditions. This general impairment does not allow any links to be drawn between specific deficits. However, without knowing the patients’ pre-stroke arithmetical abilities, we cannot rule out the possible role of pre-existing individual differences. It is widely acknowledged that arithmetic ability varies significantly between individuals. For example, Cockcroft [57] found that the average British class of 11-year-olds had, on average, a range of arithmetical abilities that were equivalent to a 7-year difference. Twenty years later, despite the introduction of the National Curriculum resulting in more standardized teaching and a mathematics syllabus, the same significant differences were found [58].

Adults also show this variability. Deloche et al. [59] administered basic numerical tests to typical adults and found substantial individual differences within a fairly homogenous sample.

Information about the extent of individual differences in arithmetic before damage was not obtainable. Pre-stroke data, as would be expected, had not been collected and therefore no information about pre-stroke arithmetical abilities was available. For example, patient R might possibly have been better at mathematics than patient P, even before their respective strokes.

Individual differences in arithmetic have been linked to individual structural differences in healthy individuals. Li et al. [60], found individual structural differences in the left inferior parietal area, which correlated with the arithmetic scores of school children. They used a variety of methods to analyze the brain area and found a structure–performance correlation. Grey matter volumes in the left superior longitudinal fasciculus and the fractional anisotropy values for the pathways within the left inferior parietal area were correlated with arithmetic scores of school children. These results suggest that differences in brain matter volume in healthy people may contribute to the range of individual differences found in arithmetic; though much more research is needed on this topic, and it is in any case difficult to establish the direction of causation: structural brain differences could be the result, as well as the cause, of individual differences in mathematical performance and associated experiences.

### 4.3. Individual Differences in Domain-Specific Numerical Abilities versus Domain-General Cognitive Abilities

The question arises whether discrepancies between different numerical tasks in patients are most likely to reflect strengths and weaknesses in domain-specific numerical abilities or to be secondary to strengths and weaknesses in domain-general cognitive abilities such as language, spatial reasoning or working memory. This is, however, not an issue restricted to patients: there is much debate about the extent to which developmental and individual differences in different numerical tasks in people without brain damage reflect domain-general or domain-specific abilities, and the strongest evidence is for both being involved.

The strongest evidence for the involvement of domain-general abilities in the aetiology of specific task deficit in this group of patients is probably with regard to the role of language and working memory in multiplication. Interestingly, patient R appeared to struggle *only* with multiplication, as his response times during this experiment were significantly slower. It may be that multiplication tasks are carried out using different strategies to some other arithmetic tasks. Teaching of multiplication tables often emphasizes rote learning and fact retrieval more than other arithmetical operations. To answer the multiplication problems, Patient R. had difficulty with fact retrieval, due to the lack of a phonological loop as a result of Broca’s aphasia. A case study of this patient [61] showed that he compensated for poor fact retrieval with good conceptual knowledge and use of derived fact strategies.

Patient P was reported to struggle with language: he also suffered from expressive aphasia. This deficit in language could be linked with poor performance in the multiplication experiment. The rote learning of multiplication sums requires stored verbal facts to be retrieved. It has been suggested that this retrieval process is very distinct, potentially even neuroanatomically separate, from the mental manipulation of numerical quantities which is required when solving addition equations [1]. A deficit in language, rather than problems with numerical cognition, is likely to affect the retrieval of multiplication answers and may explain the specific deficit seen in patient R and may also explain some of patient P’s less specific deficits.

### 4.4. Effects of Specific Aspects of the Addition Problems

The hypotheses that RTs would be higher for ascending than descending addition problems, and that RTs would increase with the number of decades that needed to be bridged, were both supported by the results. These effects were found both in the patient group and the control group, suggesting that problems that create a greater load on working memory are more difficult, but not suggesting that this is a greater or lesser problem for patients than controls. Those patients—P, S and A—who showed impairments in addition, seemed to show them for problems with and without the need to bridge decades.

### 4.5. Sampling Issues

Drawing conclusions from this study must be done with caution due to the small samples. The control sample only consisted of 11 participants. Ideally, a control sample would be much larger to gain a more representative mean. In addition, the patient group was extremely heterogeneous. Deficits, damage, and ages ranged considerably from patient to patient. With any neuropsychological patient, brain damage varies greatly and is rarely specific or contained to a specific region, thus making it difficult to draw conclusions and comparisons between patients. However, a larger sample size would have improved the power of the study as regards overall comparisons between the patients and controls, though it is less crucial to the case reports of individual patients.

## 5. Conclusions

Results of this study did not support the hypothesis that there would be a necessary relationship in this sample between subitizing and addition performance, as shown both by the lack of significant correlations between subitizing reaction time and measures of addition performance, and, more crucially, by individuals showing marked discrepancies between the two. The results also did not demonstrate significant relationships between subitizing and multiplication, though this finding must be taken with caution due to the reduced samples.

These findings do not mean that subitizing is *not* related to arithmetic. An absence of evidence for such a relationship is not the same thing as evidence for its absence. However, it can be said that there can be discrepancies between subitizing and certain aspects of arithmetic, even if there may still be an overall relationship between them. Acquired deficits in subitizing do not automatically result in deficits in basic addition ability; nor does preserving subitizing rule out such deficits. Of course, it may be that subitizing would turn out to be more strongly related to some other arithmetical abilities that were not tested here.

Moreover, subitizing could be a prerequisite for the development of numerical abilities, but not for their preservation once they have developed. Thus, once arithmetic has developed, acquired deficits in subitizing may not automatically result in loss of other arithmetical abilities, even if subitizing was essential to their development in the first place.

In addition, it is possible that there is initially a unitary number concept which later splits into functionally independent components of arithmetic ability. This split, however, would need to occur at a very early age, as there is strong evidence that arithmetical ability is already componential at the age of four. For example, Dowker [62]) tested eighty, four-year-old children on a large batch of numerical tests to investigate their understanding of math. She found, by this age, before formal instruction, that numerical ability was already divisible into components. There were no strong correlations between abilities on the different tasks. Evidence appears to converge on a componential view of arithmetic. However, it is still possible that subitizing ability may, in early years, be essential for the development of addition, which later becomes functionally independent of subitizing.

There is indeed increasing evidence that numerical abilities are more componential and functionally separable, even from infancy, than was at one time thought. Debates about whether subitizing or the approximate number system have primacy have largely given way to a view that there are two systems, one for smaller numbers and one for larger numbers. Many studies have suggested that in slightly later development, there may be a less strong relationship between symbolic and nonsymbolic aspects of numerical understanding than was at one time thought, leading to the concept of ‘symbolic estrangement’ [63]. Perhaps, just as the study of patients was important in developing our understanding of dyscalculia and the functional independence of certain numerical abilities in the first place, it will become increasingly important in refining this understanding and developing our awareness of nuances, whereby specific abilities such as subitizing may be selectively impaired, but this may not necessarily result in global mathematical impairment; and particular brain areas are strongly associated with mathematics, but damage to them does not *always* result in dyscalculia.

In this context, it must be pointed out that, as stated previously, the patients in this study were not specifically selected for having dyscalculia; nor did most have parietal lesions. While both subitizing deficits and addition deficits did occur in this sample, they may often have been secondary to deficits in other areas such as attention, working memory, speech, and motor abilities. It is possible that a sample of patients selected for mathematical deficits or for parietal lesions might have shown a closer relationship between subitizing and arithmetic. This, however, is not necessarily the case. Cappelletti et al. [46] found that most patients with selective brain lesions had some arithmetical impairment, but intact quantity processing skills, whether their lesions involved the parietal lobes or not.

The results also suggest that tests of subitizing may be only of moderate use in screening for mathematical disabilities or low mathematical attainment [41]. They are likely to be a useful tool, but only in combination with other tools. While, as discussed in the Introduction, most studies suggest that subitizing deficits occur in and probably contribute to at least some cases of developmental dyscalculia, they are far from the only factor, and many individuals with developmental dyscalculia do not have such deficits. The present study suggests that the same may be true with regard to acquired deficits in mathematics.

It is important when drawing conclusions to be clear about what the study can show. Its purpose was not to find an exact level of relationship between subitizing and specific aspects of arithmetic. The sample is too small and diverse to be able to establish the extent, if any, of such relationships; and it is likely that other factors such as age and education may influence them. Future studies should include more patients; and in particular should include larger numbers of controls with the aim of standardizing the performance levels.

The study is also not intended to find relationships between specific aspects of number understanding and specific areas of the brain, if indeed this is truly possible. Future studies should investigate this further by combining lesion studies with brain imaging studies. For example, the use of a variety of numerical tests with patients with mathematical difficulties associated with parietal lesions might help to understand whether their deficits are predominantly in subitizing or in arithmetic as such, or whether individual patients may differ in this respect. Also, the use of transcranial magnetic stimulation to produce short-term ‘virtual lesions’ in healthy volunteers may be useful in elucidating the relationships between brain areas and specific abilities, and possibly in gaining a greater understanding of the level of functional dependence or independence of different abilities, with potential implications for diagnosis and rehabilitation in clinical practice. The use of functional MRI in healthy volunteers might also help to improve our understanding of these issues. Such techniques should also provide useful additional data in patients, although the BOLD signal in fMRI may be altered by the presence of an injury in the brain parenchyma.

What the present study was intended to investigate is the extent to which individual patients may show discrepancies between subitizing and arithmetic. It shows that they may indeed do so to quite a marked degree.

## Figures and Tables

**Table 1:** Summary of the clinical and demographic details of the patient group.

Participant	Age	Sex	Handedness	Aetiology	Side of Lesion	Initial Impairments	Chronic Clinical Features
P	40	M	R	Stroke	R	Aphasia.	Expressive aphasia
R	32	M	R	Stroke	R	Aphasia	Expressive aphasia
L	44	F	L	Stroke	R	Reading. Executive	Writing. Executive
S	60	M	R	Stroke	R		_
J	63	M	R	Stroke	L	Left neglect.	No cognitive deficit detected
N	56	M	R	Stroke	L	Left neglect	Left neglect
M	73	M	R	Stroke	L	No cognitive deficit detected	No cognitive deficit detected
E	63	M	R	Stroke	L	Reading. Executive.	Rule-finding.
A	80	F:	R	Posterior Cortical Atrophy	Both_	_Number processing. Gesture imitation. Memory. Executive	Picture naming. Non-word reading. Mobility (unable to write),

**Table 2:** A summary of descriptive data for the control group in each condition.

Ability	Average	Standard Deviation
**Subitizing speed step-size**	97.21	42.58
**Subitizing accuracy %**	98.08	1.87
**Counting accuracy %**	92.35	7.22
**Counting RT slope**	304.79	111.61
**Addition RT (ms)**	2498.70	822.75
**RT with no bridge (ms)**	1895.79	602.44
**RT for one decade bridge (ms)**	2359.57	862.03
**RT for two-decade bridge (ms)**	3175.43	1033.53
**RT for ascending sums (ms)**	2560	895.06
**RT for descending sums (ms)**	2411.97	765.31
**Addition accuracy %**	93.11	3.73

**Table 3:** A summary of patients’ performance on some key measures.

Participant	Subitizing % Correct	Subitizing Step Size	Counting % Correct	Counting RT Slope	Addition % Correct	Addition Mean RT (ms) for Correct Answers	Multiplication % Correct	Multiplication Mean RT (ms) for Correct Answer
P	97.77	210.38	96.67	1210.38	85.71	23,572.05	_	_
R	98.89	113.74	97.5	228.79	95.31	4399.08	87.5	4082.24
L	100	89.6	94.17	406.29	96.87	3850.16	_	_
S	98.8	85.48	97.5	390.62	83.58	4658.2.	_	-
J	100	47.52	95.83	283.52	96.88	1704.04	100	931.56
N	98.89	864.44	77.5	781.57	88.28	27,443.33	89.66	2055.42
M	100	6.29	94.17	273.6	94.53	2504.89	_	_
E	92.22	113.19	86.67	463.05	92.19	2406.3	98.46	1779.59
A	86.67	−60.5	20.83	32,411.88	78.57 *	_	62.16	29,589.82
Controls (mean)	98.24	95.78	92.22	343.25	91.41	2614.2	89.29	1775.28
Controls (S,D)	1.86	40.9	6.9	170.5	6.89	880.69	3.84	604.48

* A was given a reduced set of 14 addition sums.

**Table 4:** A summary of the significant impairments seen in patients relative to controls.

Patient	Impaired Abilities	Average	Standard Deviation	Crawford’s t	*p* Value
***P***	Subitizing speed step-size	216.38	_	2.679	0.023
***P***	Addition RT	23,572.41	23,775.49	24.523	<0.001
***P***	RT with no bridge	12,770.5	7128.43	17.283	<0.001
***P***	RT with one-decade bridge	14,493.23	7962.89	13.476	<0.001
***P***	RT with two-decade bridge	44,412.13	29,049.66	−38.201	<0.001
***P***	RT ascending	25,808.43	21,376.98	24.867	<0.001
***P***	RT descending	20,714.44	26,646.83	22.896	<0.001
***P***	Accuracy with no bridge	80	--	−3.307	0.008
***R***	Multiplication RT	4082.84	3814.61	3.571	0.012
***S***	RT to Addition with no bridge	4143.00	1703.71	3.572	0.005
***S***	RT with one-decade bridge	4587.78	1204.69	2.475	0.033
***S***	RT descending	4629.88	1158.77	2.775	0.02
***S***	Accuracy with one-decade bridge	68.57	_	−3.75	0.004
***S***	Accuracy descending	77.19	_	−2.552	0.029
***N***	Subitizing speed step-size	864.45	_	17.251	<0.001
***E***	Subitizing accuracy	92.22	_	−3.008	0.013
***A***	Subitizing accuracy	86.67		−8.86	<0.001
A	Counting accuracy	20.86	_	−9.477	<0.001
A	Accuracy for addition with no bridge	81.82	_	−2.888	0.016
A	Accuracy with one bridge	66.67	_	−6.134	<0.001
	Multiplication RT	29,589.82	22,794.35	43.043	<0.001
	Multiplication accuracy	62.16	_	−6.552	<0.001

Patients L, J and M all performed within the control participants’ range for all tasks.

## Data Availability

Data requests can be made to ann.dowker@psy.ox.ac.uk.
